# Sex Specificities in the Association Between Diet, Physical Activity, and Body Composition Among the Elderly: A Cross-Sectional Study in Florence, Italy

**DOI:** 10.3390/ijerph22070975

**Published:** 2025-06-20

**Authors:** Nora de Bonfioli Cavalcabo’, Luigi Facchini, Melania Assedi, Ilaria Ermini, Flavia Cozzolino, Emma Bortolotti, Calogero Saieva, Davide Biagiotti, Elisa Pastore, Benedetta Bendinelli, Giovanna Masala, Saverio Caini

**Affiliations:** 1 Cancer Risk Factors and Lifestyle Epidemiology Unit, Institute for Cancer Research, Prevention and Clinical Network (ISPRO), 50139 Florence, Italy; n.debonfiolicavalcabo@ispro.toscana.it (N.d.B.C.); l.facchini@ispro.toscana.it (L.F.); m.assedi@ispro.toscana.it (M.A.); i.ermini@ispro.toscana.it (I.E.); f.cozzolino@ispro.toscana.it (F.C.); e.bortolotti@ispro.toscana.it (E.B.); c.saieva@ispro.toscana.it (C.S.); d.biagiotti@ispro.toscana.it (D.B.); g.masala@ispro.toscana.it (G.M.); 2 Clinical Epidemiology Unit, Institute for Cancer Research, Prevention and Clinical Network (ISPRO), 50139 Florence, Italy; e.pastore@ispro.toscana.it (E.P.); b.bendinelli@ispro.toscana.it (B.B.)

**Keywords:** dietary patterns, physical activity, anthropometry, body composition, elderly, sex differences

## Abstract

The rising prevalence of elderly obesity in developed countries poses a public health challenge, since body composition changes during aging are associated with higher risks of chronic diseases. We cross-sectionally explored the relationship between diet, physical activity, and sex-specific differences in body composition among 378 elderly previously enrolled in the Florence European Prospective Investigation into Cancer and Nutrition (EPIC) cohort. Information on dietary habits and lifestyle was collected through validated questionnaires. Adherence to the Italian Mediterranean Index (IMI), Dietary Approaches to Stop Hypertension (DASH), and Greek Modified Mediterranean Diet (GMMD) a priori dietary patterns was calculated. Anthropometric measures were taken by trained personnel, and body composition parameters were estimated via bioelectrical impedance. In age- and energy-intake-adjusted regression models, adherence to the DASH and IMI patterns was associated with healthier body composition among women, while no significant relationship emerged among men. Fitness activities and total recreational physical activity revealed positive associations with healthier body composition (lower % fat mass, higher % muscle mass, and reduced waist circumference) in both sexes. These findings highlight the synergistic effect of diet and physical activity on body composition in the elderly and underscore the need for sex-specific interventions for promoting healthy aging.

## 1. Introduction

Elderly obesity has become a major public health concern in developed countries with aging populations. Weight gain in adulthood results from a combination of non-modifiable (aging, metabolism alteration, menopause) and modifiable factors, resulting in an imbalance between calories consumed and calories expended. The global epidemic of elderly obesity parallels the rise in the incidence of so-called “adiposity-based” chronic diseases (including cancer, diabetes, and cardiovascular disease), i.e., diseases for which excessive body fat accumulation is an established risk factor and that rank among the major causes of death globally [1,2]. The focus on obesity is a consequence of both its high prevalence and strong association with multiple health outcomes and the possibility of modifying it through lifestyle changes at any age in life, thus making it an ideal target for public health interventions.

Sex is a relevant aspect for the effect of obesity on chronic diseases because the differences between men and women are not only limited to the total percentage of body fat but extend to its body distribution [3]. Men tend to accumulate abdominal visceral fat, while pre-menopausal women mostly gain fat in their hips and thighs. After menopause, this difference becomes more nuanced, as adipose tissue is redistributed in the abdomen also among women [2]. Along with aging, body composition undergoes changes that are partly independent from physiological fluctuations in weight and body mass index (BMI) [4]. These changes are characterized by a progressive decrease in fat-free mass (body water, muscle, and bone mass) and a concomitant increase in fat mass; a redistribution of fat mass toward the trunk; an increased infiltration of fat in organs such as the liver and muscles; and a reduction in body height and bone density [5]. These variations are common to both sexes in older age, yet body composition continues to differ between sexes, since the percent fat mass remains higher in women than men irrespective of any changes in total body adiposity [6]. Furthermore, it is essential to underline how biological sex is one of the major determinants of the burden of most chronic–degenerative diseases related to overweight and obesity, including cardiovascular diseases, cancer, and dementia, further emphasizing the importance of taking it into consideration both in research and in planning primary prevention interventions.

BMI is a useful measure of adiposity as it captures the component of weight that is independent of body height, but it fails to discriminate between muscle and fat mass and to identify where body fat is located, a most important piece of information given the stronger association of disease with visceral fat in the abdomen rather than peripheral fat. Partially overcoming these limitations, waist circumference (WC) is an indicator of abdominal obesity currently recommended as the most appropriate measurement of obesity in postmenopausal women. However, WC has drawbacks too, e.g., its inability to differentiate subcutaneous from visceral fat deposition [7,8,9]. Bioelectrical Impedance Analysis (BIA) is a more accurate method for assessing body composition, is essential for the characterization of metabolic status in the elderly, and might offer the opportunity to provide sex-specific and personalized advice and counseling [4]. Extensive evidence underlines the importance of a detailed assessment of body composition and its relationship to disease risk. For example, Drozdová et al. found that middle-aged Slovakian women suffering from cardiovascular complications had significantly altered electrical tissue properties and higher adiposity indices (BMI and WHR) compared with healthy controls, suggesting a close relationship between CVD and body composition [10]. Similarly, Vorobelová et al. reported significant differences in muscle mass and total body water between metabolically healthy and unhealthily obese women, indicating the complexity of metabolic phenotypes in midlife [11]. Complementing these findings, a systematic review by Kim et al. (2022) showed that age-related changes in muscle and fat distribution have different effects on health in men and women [12]. While skeletal muscle mass (sMM) tends to benefit both sexes, the protective effects are significantly weaker in women. Conversely, fat mass (FM) appears to be more detrimental to physical performance in women and shows different associations with metabolic and cognitive outcomes depending on anatomical location and gender. These sex-specific physiological responses to changes in body composition justify the need for targeted interventions in aging populations. 

Adoption of a healthy lifestyle is a first-line option to tackle mid- and late-life changes in body adiposity and maintain or restore a correct body composition. In addition, lifestyle habits (e.g., dietary habits and exercise) are well-known for their association with countless health outcomes, including cardiovascular diseases and cancer, even when changes are made in older age [13,14,15,16]. Again, it is key for sex to be brought into the picture, given its known association with the prevalence of lifestyle habits and their lifetime changes. What remains relatively poorly understood is the complicated network of interactions between sex, lifestyle (particularly diet and physical exercise), and body composition in elderly individuals. This is a particularly severe knowledge gap as it has the potential to undermine the effectiveness of health interventions and the possibility of tailoring them to the specific needs of each individual. The present cross-sectional study seeks to help fill this knowledge gap by exploring, in a group of individuals of both sexes in their late adulthood and older age living in Tuscany (central Italy), the relationship between physical activity (PA) levels and adherence to a priori dietary patterns, with body composition measures including BMI and WC as well as BIA-derived parameters including percent fat, muscle, and bone mass, visceral fat, total body water, and percent intra- and extracellular water.

## 2. Materials and Methods

### 2.1. Study Population and Data Collection

The study that originated the data presented in this article was approved by [removed for blind peer review]. This study was carried out in a group of elderly individuals previously enrolled in the Florence section of the European Prospective Investigation into Cancer and Nutrition (EPIC) cohort. The EPIC study and its methodology are described in detail elsewhere [17]. Briefly, information on lifestyle and diet was collected from 13,597 volunteers residing in Florence between 1993 and 1998. Data on smoking, alcohol intake, education, occupation, physical activity, and medical and reproductive history were gathered by means of a validated sex-specific lifestyle questionnaire (LSQ). Detailed information on dietary intakes was collected through a validated food frequency questionnaire (FFQ) specifically developed to capture Italian dietary habits [17,18]. Dietary data from the FFQ were converted into daily food intake by using software specifically developed within the EPIC study; then, caloric intake (in kcal or kJ) was calculated by using the Food Composition Database for Epidemiological Studies in Italy (BDA) developed at the European Institute of Oncology (IEO, Milan, Italy) [19]. Weight, height, hip and waist circumference, blood pressure, and heart rate were measured by trained health personnel. Hip circumference was measured at the fullest part of each participant’s hips, while waist circumference was measured horizontally after locating the midpoint between the bottom of the ribs and the top of the hips. A fasting venous blood sample was taken from each participant, divided into plasma, red-cell, and buffy-coat aliquots, and stored in the project biobank.

The present investigation was conducted within the Epimetal research project, whose overarching objective is to improve our understanding of the public health relevance of heavy metal contamination in Tuscany and whose investigations (including those not presented here) have been mostly conducted among individuals who enrolled in the EPIC Florence cohort in the 1990s. The protocol of the Epimetal research project envisaged reaching a sample size of 300 participants; however, the enrollment was extended beyond what was initially planned given the availability of time and resources, with the aim of further increasing the statistical power of any investigations conducted within it. Thus, a total of 650 individuals randomly selected from the EPIC Florence cohort were invited to join the Epimetal project in 2021, and 378 agreed to participate (participation rate 58.2%). Compared with participants, those who declined were significantly older (mean age 79.5 vs. 74.4 years, *p*-value < 0.001); moreover, the participation rate was significantly higher among men (158/227, 69.6%) than women (220/423, 52.0%). The enrollment procedure consisted of a follow-up outpatient visit, during which information on lifestyle and diet and anthropometric measurements were updated, and body composition measures were collected for the first time by means of a BIA body composition analyzer. The present analysis was limited to 325 participants who could go to the study facilities and for whom it was possible to collect information on their body composition (53 were visited at home because of illness or disability).

Physical activity habits (including occupational, household, and recreational activities) of all participants were updated through the EPIC self-administered LSQ (checked by trained personnel during the follow-up visit) [20,21]. Volunteers first had to declare if they currently had a paid full- or part-time job and to classify it as sedentary, standing, manual, or heavy manual. They were then requested to indicate the weekly hours spent in leisure-time activities in summer and winter, in particular walking, cycling, gardening, and fitness activities (e.g., gym activities, swimming, and running). Participants were also requested to mark the number of weekly hours of do-it-yourself activities, the daily number of hours spent in housework, and the number of floors of stairs climbed per day. The energy expenditure corresponding to each activity type was measured using the standard Total Metabolic Equivalent (MET) hours/week [22]. Finally, an overall “total physical activity index” (in four categories: inactive, moderately inactive, moderately active, and active) was derived based on occupational and leisure-time physical activity [21,22]. The full details on how the physical activity variables were derived from the data collected in the EPIC lifestyle questionnaire are available in Ainsworth BE et al. [22].

Body composition was evaluated within the Epimetal research project via the TANITA MC-780MA bioelectrical impedance body composition analyzer. The instrument consists of a stand-alone unit where the age, sex, and height of participants are inputted by the operator. During the measurement, participants must step on a special platform with bare feet and take the two handles with both hands alongside the body; then, participants’ weight, percent fat, muscle, and bone mass, visceral fat, total body water, and percent intra- and extracellular water are estimated [23]. Height was measured using a stadiometer and BMI was calculated as weight (kg)/height^2^ (m). Waist and hip circumferences were obtained using a tape measure.

Eating habits were updated by means of the self-administered EPIC FFQ, which was also checked by the study personnel during the follow-up visit. Respondents were asked to indicate, referring to the previous year, their average frequency of food consumption (number of times per week or per month or year in the case of less frequent consumption) and the typical portion size of each food. The questionnaire covers a wide range of foods, including grains, soup, meat, fish, raw and cooked vegetables, dairy products, sandwiches, salami and other cured meats, fruit, bread, cakes/sweets, herbs/spices, and beverages. The quantity of the food consumed was estimated using photographs of standard portion sizes or household measures [24]. A specific software program was used to convert dietary data from the questionnaire into average daily amounts of foods (g/day). Nutrition analysis of the FFQ was linked to the Italian Food Composition Tables (FCTs) for nutrients and energy assessment [16].

### 2.2. A Priori Dietary Patterns

We investigated the adherence of all study participants to three a priori-defined diet patterns, namely the Italian Mediterranean Index (IMI), the Dietary Approaches to Stop Hypertension diet (DASH), and the Greek Modified Mediterranean Diet (GMMD).

The IMI was developed to assess the adherence to the Italian Mediterranean Diet and includes eleven items, of which six are typical Mediterranean foods (pasta, local vegetables and fruit, legumes, olive oil, and fish) and five components not typically present in the Mediterranean diet (soft drinks, alcohol, butter, red meat, and potatoes) [25]. A value of 0 or 1 is assigned to each of the 11 components; 1 point is attributed if consumption of typical Mediterranean foods is in the third tertile of the distribution, all other intakes receiving 0 points. If consumption of non-Mediterranean foods is in the first tertile of the distribution, the person also receives 1 point; otherwise, 0 points are assigned. IMI scores range from 0 to 11.

To assess the degree of adherence to the DASH diet, we scored eight foods, including presumed beneficial components (fruits, vegetables, nuts and legumes, low-fat dairy products, and whole grains) and presumed harmful components (sweetened beverages, sodium, and red and processed meat) [26]. High scores indicated high consumption of beneficial components and vice versa. The scores (from 1 to 5) assigned to each food are then summed up to obtain the total DASH score, which ranges between 8 and 40 points.

The GMMD is based on the Mediterranean diet scale [27]. Scoring is based on intake of nine items: vegetables, legumes, fruit and nuts, dairy products, cereals, meat and its derived products, fish, alcohol, and the ratio of unsaturated (sum of mono- and polyunsaturated) to saturated fat. Persons whose consumption of presumed beneficial components is below the median consumption among the study participants are assigned a value of 0, whereas for individuals with consumption above the median a value of 1 is given, and inversely for presumed harmful components. For ethanol, a value of 1 was given to men consuming from 10 g to less than 50 g of it per day; for women, the corresponding cut-offs were from 5 g to 25 g/day. This score ranges from 0 (minimal adherence) to 9 (maximal adherence).

### 2.3. Statistical Analysis

The distribution of continuous and categorical variables was compared between sexes by using the *t*-test and exact Fisher’s test for continuous and categorical variables, respectively (normality of distribution was checked via QQ plots and the Kolmogorov–Smirnov test). We fitted linear regression models to investigate the association between adherence to the predefined dietary patterns or recreational and household physical activity levels (explanatory variables) with each of BMI, waist circumference, and visceral fat score (response variables), which were entered into the models as continuous variables. Beta regression models with the same explanatory variables were fitted for the four response variables that were expressed as percentages (i.e., % fat, muscle, and bone mass and % total body water). Of note, we did not use occupational physical activity as an explanatory variable because of the well-known “physical activity paradox”, according to which occupational physical activity is usually not clearly associated with health benefits and may even be harmful [28]. The variables quantifying adherence to each a priori dietary pattern were entered into the models after splitting them into three predefined categories (0–2, 3–4, and 5–9 for IMI; ≤23, 24–27, and ≥28 for DASH; and 0–3, 4–5, and 6–8 for GMMD), always considering the bottom category (indicating minimal adherence) as the reference category. Each physical activity variable was entered into the models either as a continuous variable (reporting the coefficient for a +1 MET hours/week increment) or in quartiles (showing the coefficient for the comparison of the top vs. bottom quartile; tertiles were used for fitness activities due to their narrower distribution). The linear or beta regression models for the seven response variables were all fitted separately among men and women and adjusted by age and caloric intake (entered in the models as cubic splines for finer adjustment). Adding further adjustment variables did not substantially change the coefficients of the explanatory variables, and they therefore were not included in the final models. The analyses were conducted by using Stata software, version 17. All statistical tests were two-sided, and statistical significance was set at a *p*-value below 0.05.

## 3. Results

The distribution of the main sociodemographic characteristics at Epimetal follow-up of the 325 EPIC-Florence participants (142 men, 43.7% and 183 women, 56.3%) is reported in Table 1. All participants were over 59 years old with a mean age of 73.2 (standard deviation (SD) 5.7, range 62–88) years for men and 72.8 (SD 6.4, range 59–88) years for women. Only a minority of participants were active smokers (12.0% of men and 8.2% of women), while former smokers made up the larger group among both sexes. The proportion of participants still working was 23.2% and 16.6% among men and women, respectively.

Men performed more recreational activities than women (34.0 vs. 28.5 MET hours/week, *p*-value 0.060), while women spent three times as much time as men engaged in physical activity at home (*p*-value < 0.001) (Table 2). The time spent by men in walking and fitness activities was close to that of women. A much larger proportion of men were classified as active or moderately active compared with women (52.1% vs. 26.2%), while the proportions of those classified as inactive were only slightly different (7.7% vs. 11.5%).

Men had a slightly higher BMI than women on average (27.3 vs. 26.3 kg/m^2^, *p*-value 0.003), and a larger proportion of men were classified either as overweight (≥25 to <30 kg/m^2^: 50.0% vs. 35.0%) or obese (≥30 kg/m^2^: 22.5% vs. 19.7%) (Table 2). The average waist circumference was also significantly larger among men, but the proportion of participants exceeding the 102 or 88 cm threshold (for men and women, respectively) did not differ between sexes. Many differences between sexes were observed with regard to body composition measures. Upon adjusting by age and total caloric intake, women were characterized by having significantly higher percent fat mass (34.1% vs. 23.5%) and lower percent muscle (62.5% vs. 72.7%) and bone mass (3.4% vs. 3.8%) as well as a lower proportion of total body weight made up of water (46.2% vs. 52.7%). Moreover, the total body water was to a greater extent in the cellular compartment in men than women (55.4% vs. 52.6%, *p*-value < 0.001) (Table 2).

### 3.1. Dietary Patterns and Body Composition

The association between the adherence to each of the three a priori dietary patterns is shown in Table 3. We found that a much larger proportion of men (39.4%) and women (40.4%) fell into the highest IMI score category (“good adherence”) compared with those in the “low adherence” category (respectively 25.4% and 20.8%). The opposite was seen for the DASH pattern, where the largest proportion of men (41.5%) and women (53.5%) were labelled as poorly adherent (≤23), while for GMMD the most represented category was the middle one (score 4–5) among both sexes.

No significant association emerged between adherence to the three dietary patterns and any of the body composition parameters considered among men (Table 3). Among women, scoring ≥28 (“good adherence”) vs. ≤23 (“low adherence”) in the DASH pattern was inversely associated with percent fat mass (β −3.65%, *p*-value 0.006), visceral score (β −1.36, *p*-value 0.006), BMI (β −2.77, *p*-value 0.005), and waist circumference (β −7.32, *p*-value 0.002) and directly associated with percent muscle mass (β 3.42%, *p*-value 0.006), bone mass (β 0.17%, *p*-value 0.010), and total body water (β 2.39%, *p*-value 0.008). When IMI was considered, “good adherence” (score 5–9) as compared with “low adherence” (score 0–2) was associated with a significantly lower BMI (β −2.12, *p*-value 0.027) and waist circumference (β −4.97, *p*-value 0.035). No significant association with any anthropometric or body composition parameter emerged for adherence to GMMD.

### 3.2. Physical Activity Levels and Body Composition

The associations between physical activity levels and anthropometric and body composition characteristics are reported in Table 4 and Table 5 for men and women, respectively. In models adjusted for total caloric intake and age, higher levels of fitness activities and overall recreational physical activity were associated with a healthier body composition profile among both sexes, including a lower body fat percentage, a higher muscle mass percentage, a lower visceral fat score, and a smaller waist circumference. Instead, neither among men nor women did we observe significant associations between walking and household physical activity and body composition parameters.

Regarding men, recreational physical activity showed a direct association with percent bone mass (β 0.133%, *p*-value 0.039) and an inverse association with BMI (β −1.58, *p*-value 0.046) and WC (β −5.36, *p*-value 0.025) when comparing those in the fourth vs. first quartile (Table 4). Men in the third tertile of fitness (sports activities with MET values ≥ 6) showed statistically significant inverse associations with percent fat mass (β −3.29, *p*-value 0.008), visceral fat score (β −1.99, *p*-value 0.004), BMI (β −2.31, *p*-value 0.004), and waist circumference (β −7.24, *p*-value 0.02) than men in the first tertile, showing instead a direct association with % muscle mass (β 3.10%, *p*-value 0.007), bone mass (β 0.176%, *p*-value 0.004), and total body water (β 2.11%, *p*-value 0.014). When modeled as continuous variables, both fitness and overall recreational physical activity were significantly positively associated with % muscle mass, bone mass, and total body water and inversely associated with % fat mass, visceral fat score, BMI, and waist circumference (Table 4). For the total physical activity index, men labeled as “moderately active” or “active” had a significantly smaller waist circumference (β −8.69, *p*-value 0.023) compared with those in the “inactive” category.

Among women, both fitness activities and overall recreational physical activity were significantly associated with a healthier body composition (i.e., lower % fat mass and visceral fat score and higher % muscle and bone mass and total body water) and reduced BMI and waist circumference, both when comparing women in the highest vs. lowest distribution quantile and when modeling physical activity as continuous variables in the models (Table 5). Finally, no associations emerged among women among the total physical activity index and body composition or anthropometric parameters. In general, the β coefficients tended to be slightly farther from the null value among women than among men, except for waist circumference, where the opposite trend was observed, but these differences were never statistically significant (i.e., the *p*-value for interaction was always >0.05).

## 4. Discussion

In the present cross-sectional study, we explored the impact of physical activity and diet on body composition parameters in elderly individuals of both sexes residing in Florence and the surrounding area. Women and men in the study sample differed in many regards, with the latter being generally more active (except for energy spent in housekeeping), more frequently overweight or obese, but less often exceeding the waist circumference threshold. In terms of body composition, men had a generally larger percentage of body weight represented by muscle mass, bone, and intracellular water, while fat mass and extracellular water made up a larger share of body weight among women. Concerning diet, our study population demonstrated good adherence to the IMI pattern but poor adherence to the DASH and GMMD patterns, especially among women.

In terms of the effect of physical activity and diet on body composition parameters, we found that participation in recreational physical activity (especially when intense, e.g., sport activities) and, limited to women, adherence to healthy dietary patterns were associated with significantly improved body composition parameters. In particular, among women, good adherence to both the IMI and DASH diets was associated with significantly lower BMI and waist circumference, while only the latter was also associated with healthier body composition (significantly lower % body fat, higher % muscle mass, bone mass, and total body water, and significantly lower visceral fat score, BMI, and waist circumference). The lack of an association between diet and body composition among men might be due to the smaller sample size (since the association, while not statistically significant, was mostly in the expected direction) or different dietary habits not fully caught by predefined patterns. We recently obtained similar results on the IMI pattern in a group of 388 postmenopausal women in a cross-sectional study also nested within the EPIC-Florence cohort, where higher adherence to the pattern was inversely associated with BMI, waist circumference, and % fat mass and directly associated with % muscle mass. In the same study, higher levels of recreational PA were associated with lower percent fat mass, BMI, and waist circumference and higher percent muscle mass values [29]. In the literature on the topic, the results were not always consistent with ours, especially when it comes to sex-stratified analyses. A recent investigation from France reported a significant association between adherence to a “Mediterranean-like” diet and BMI, body fat, and lean mass, although (unlike our study) limited to middle-aged men [30]. A US study encompassing over 3000 older adults also found men to benefit more than women (in terms of body composition parameters) when adhering to a “health food” pattern (although the association was modified by the peroxisome-proliferator-activated receptor-c (PPAR-c) gene status) [31], while in a previous study also from the US an association in this direction was seen mostly among women [32]. In general, comparability with previous publications was made difficult by the broad diversity in terms of populations’ dietary habits and the patterns (either a priori or a posteriori) that were used in the aim to capture the participants’ eating style.

In terms of physical activity, the total amount of recreational physical activity and the time spent in fitness activities were consistently associated with normal weight and better body composition among both sexes. However, walking or household activity showed no significant association, highlighting the importance of regular and moderate-to-intense physical exercise (such as brisk walking, cycling, and other fitness activities) in positively influencing body composition parameters. The potential of regular physical exercise in maintaining a healthy body composition during aging has mostly been confirmed by the literature on the topic [33,34,35]. For instance, higher levels of total leisure-time physical activity were consistently associated with reduced incidence of abdominal and general obesity in the framework of the Prevention with Mediterranean Diet (PREDIMED) prospective cohort study, i.e., in a population reasonably similar, in terms of dietary pattern and lifestyle habits, to that included in the present study [36].

The strengths of this study include the availability of direct anthropometric and body composition measurements and the accurate collection of dietary and lifestyle data obtained by means of validated questionnaires in a sample of elderly individuals previously enrolled in a general-population-based prospective cohort study. The analysis of body composition was performed using the TANITA MC-780MA (TANITA, Tokyo, Japan) analyzer, which is commonly regarded as an adequate method for use in epidemiological studies among individuals of both sexes in their late adulthood and older age [37,38]. In this regard, it is important to point out that, in our analysis, we opted to include visceral fat score among the body composition parameters under study, although this is not measured directly (unlike the % of fat and muscle mass) but rather estimated via an indirect algorithm by the TANITA instrument. The large amount of data originating from the EPIC food-frequency questionnaire was exploited by using adherence to dietary patterns in the analysis. These examine the effect of overall diet, which can be more predictive of future disease risk than individual foods or nutrients. Our study also has limitations that need to be fully acknowledged, the most important being its cross-sectional design (a consequence of the fact that information on body composition using BIA was not collected at cohort inception in the 1990s, but only in 2021–2022 within the Epimetal project), which prevents us from studying cause–effect chains with the scientific validity guaranteed by prospective studies. However, the biological plausibility of the associations studied and the typically gradual modification of the individual characteristics under study (diet, physical exercise, and body composition) among the elderly contribute to the credibility of our results. The differential participation rate by demographic (age and sex) might have introduced selection bias (especially among older women), whose impact on generalizability is difficult to determine. Moreover, a subset of those who accepted to join the Epimetal project were visited at home, mostly because of an underlying illness or disability that prevented them from travelling to the project facility. These individuals were not included in the present investigation due to the lack of data on body composition, which further cautions against extrapolating our results to older individuals with major comorbidities. Finally, as in all observational epidemiological studies, reliance on self-reported dietary and physical activity data may result in potential recall bias and misclassification, whose impact on our findings is difficult to predict. However, the very detailed and validated EPIC questionnaires were found to provide a robust foundation for analysis [17,18,20,21].

## 5. Conclusions

In summary, our study sheds light on sex-based differences in dietary patterns, the intensity and amount of physical activity, and body composition among elderly Italians. It underscores the importance of diet and physical activity in maintaining a healthy BMI and waist circumference, particularly influencing body composition for the combination of both factors, especially among women. Our findings reinforce the notion that a balanced lifestyle represents a viable option to positively affect the risk of a broad spectrum of health conditions in older people. In fact, studies have established a strong link between body composition and the risk and clinical course of several chronic diseases as well as poorer overall and cause-specific survival rates [37,38,39,40,41,42]. Thus, public health interventions aimed at promoting healthy aging should consider tailoring strategies to the distinct needs of both women and men, emphasizing the significance of adopting a balanced lifestyle to enhance body composition and prevent chronic diseases. Future research directions in this area could include a prospective longitudinal approach aimed at assessing the role of body composition (and changes therein) in mediating the relationship between diet and physical activity levels (including changes occurring in older age) and major health outcomes.

## Figures and Tables

**Table 1:** Distribution of sociodemographic characteristics and smoking status among 142 male and 183 female EPIC-Florence cohort participants included in the present investigation.

	Men (n = 142)		Women (n = 183)		*p*-Value
	N/Mean	%/SD	N/Mean	%/SD	
**Age, years**	73.2	5.7	72.8	6.4	0.509
**Marital status**					
Married/living as married	109	77.9%	107	59.4%	
Widowed	13	9.3%	37	20.6%	
Divorced or separated	11	7.9%	18	10.0%	
Never been married	7	5.0%	18	10.0%	0.004
**Education, highest level attained**					
None, primary, or lower secondary school	45	31.9%	50	27.6%	
Professional or upper secondary school	62	44.0%	79	43.6%	
University degree or above	34	24.1%	52	28.7%	0.569
**Smoking status**					
Never smoker	51	35.9%	83	45.4%	
Former smoker	74	52.1%	85	46.4%	
Current smoker	17	12.0%	15	8.2%	0.182
**Occupational physical activity**					
Unemployed	109	76.8%	151	83.4%	
Sedentary occupation	21	14.8%	21	11.6%	
Standing occupation	4	2.8%	7	3.9%	
Manual or heavy manual occupation	8	5.6%	2	1.1%	0.088

Numbers may not sum up to totals due to missing values (the proportion of missing values did not exceed 2% for any variable).

**Table 2:** Distribution of physical activity levels and anthropometric and body composition measures among 142 male and 183 female EPIC-Florence cohort participants included in the present investigation.

	Men (n = 142)		Women (n = 183)		*p*-Value
	N/Mean	%/SD	N/mean	%/SD	
**Physical activity, MET hours/week**					
Recreational physical activity	34.0	29.1	28.5	23.9	0.060
*Walking*	*16.9*	*13.6*	*15.1*	*10.5*	*0.167*
*Fitness*	*10.4*	*16.0*	*9.7*	*15.2*	*0.687*
Household physical activity	19.8	23.6	57.9	37.3	<0.001
**Total physical activity**					
*Inactive*	11	7.7%	21	11.5%	
*Moderately inactive*	57	40.1%	114	62.3%	
*Moderately active or active*	74	52.1%	48	26.2%	<0.001
**Anthropometry**					
Weight (kg)	80.0	12.3	64.3	11.6	<0.001
Height (cm)	171.3	6.8	156.4	6.2	<0.001
Body mass index (kg/m^2^)	27.3	3.9	26.3	4.6	0.047
*<25*	*39*	*27.5%*	*83*	*45.4%*	
*≥25–<30*	*71*	*50.0%*	*64*	*35.0%*	
*≥30*	*32*	*22.5%*	*36*	*19.7%*	*0.003*
Waist circumference (cm)	96.9	11.2	85.2	11.4	<0.001
*< 94 (M)/< 80 (F)*	*60*	*42.3%*	*65*	*35.5%*	
*≥94–*≤ *102 (M)/≥ 80–*≤ *88 (F)*	*39*	*27.5%*	*56*	*30.6%*	
*>102 (M)/> 88 (F)*	*43*	*30.3%*	*62*	*33.9%*	0.465
Hip circumference (cm)	102.0	7.5	100.8	9.6	0.192
**Body composition (kg)**					
Fat mass	19.4	7.3	22.6	8.0	<0.001
Muscle mass	57.6	6.2	39.6	4.2	<0.001
Bone mass	3.0	0.3	2.1	0.2	<0.001
Visceral fat (score)	14.9	3.5	9.6	2.6	<0.001
Water, total body	41.8	4.8	29.2	3.2	<0.001
*Water, intracellular*	*23.2*	*3.1*	*15.4*	*1.7*	*<0.001*
*Water, extracellular*	*18.6*	*1.7*	*13.9*	*1.7*	*<0.001*
**Percent body composition (%)**					
Fat mass	23.5%	5.9	34.1%	6.7	<0.001
Muscle mass	72.7%	5.6	62.5%	6.3	<0.001
Bone	3.8%	0.3	3.4%	0.3	<0.001
Water, total body	52.7%	4.2	46.2%	4.7	<0.001
*Water, intracellular ^(a)^*	*55.4%*	*1.6*	*52.6%*	*1.9*	*<0.001*
*Water, extracellular ^(a)^*	*44.6%*	*1.6*	*47.4%*	*1.9*	*<0.001*

^(a)^ These percentages were calculated over the total body water. Numbers may not sum up to totals due to missing values (the proportion of missing values did not exceed 2% for any variable).

**Table 3:** Adherence to the Italian Mediterranean Index (IMI), Dietary Approaches to Stop Hypertension (DASH), and Greek Modified Mediterranean Diet (GMMD) a priori dietary patterns and percent body composition measures among 142 males and 183 females from the EPIC-Florence cohort.

Dietary Score	% Participants	Fat Mass, %			Muscle Mass, %			Bone Mass, %			Total Body Water, %		
		β	95% CI	*p*-Value	β	95% CI	*p*-Value	β	95% CI	*p*-Value	β	95% CI	*p*-Value
**Men (n = 142)**													
**IMI (ref: 0–2)**	25.4	ref			ref			ref			ref		
3–4	35.2	−0.26%	(−2.76; 2.23)	0.835	0.23%	(−2.11; 2.57)	0.847	0.01%	(−0.12; 0.14)	0.895	−0.07%	(−1.79; 1.66)	0.941
5–9	39.4	−0.79%	(−3.29; 1.71)	0.534	0.80%	(−1.54; 3.15)	0.502	0.05%	(−0.07; 0.18)	0.399	0.31%	(−1.42; 2.05)	0.723
**DASH (ref: ≤23)**	41.5	ref			ref			ref			ref		
24–27	30.3	0.27%	(−2.04; 2.58)	0.818	−0.19%	(−2.36; 1.98)	0.863	0.01%	(−0.11; 0.13)	0.852	−0.08%	(−1.69; 1.52)	0.919
≥28	28.2	−1.51%	(−3.93; 0.91)	0.221	1.44%	(−0.83; 3.71)	0.215	0.09%	(−0.03; 0.22)	0.138	1.05%	(−0.64; 2.74)	0.222
**GMMD (ref: 0–3)**	26.1	ref			ref			ref			ref		
4–5	40.8	0.62%	(−1.78; 3.02)	0.612	−0.54%	(−2.80; 1.71)	0.636	−0.01%	(−0.13; 0.11)	0.882	−0.52%	(−2.19; 1.15)	0.540
6–8	33.1	−0.46%	(−3.07; 2.14)	0.728	0.48%	(−1.97; 2.93)	0.701	0.06%	(−0.08; 0.19)	0.417	0.31%	(−1.49; −1.49)	0.733
**Women (n = 183)**													
**IMI (ref: 0–2)**	20.8	ref			ref			ref			ref		
3–4	38.8	−1.99%	(−4.58; 0.60)	0.132	1.84%	(−0.60; 4.27)	0.139	0.10%	(−0.03; 0.23)	0.130	1.32%	(−0.43; 3.06)	0.139
5–9	40.4	−1.51%	(−4.13; 1.11)	0.260	1.38%	(−1.08; 3.84)	0.271	0.08%	(−0.05; 0.21)	0.243	0.93%	(−0.82; 2.68)	0.299
**DASH (ref: ≤23)**	53.5	ref			ref			ref			ref		
24–27	29.0	−1.37%	(−3.63; 0.89)	0.234	1.22%	(−0.90; 3.34)	0.260	0.08%	(−0.04; 0.19)	0.181	0.78%	(−0.74; 2.31)	0.315
≥28	17.5	−3.65%	(−6.22; −1.07)	**0.006**	3.42%	(0.98; 5.85)	**0.006**	0.17%	(0.04; 0.31)	**0.010**	2.39%	(0.61; 4.16)	**0.008**
**GMMD (ref: 0–3)**	31.1	ref			ref			ref			ref		
4–5	42.1	1.32%	(−1.03; 3.67)	0.270	−1.26%	(−3.47; 0.96)	0.265	−0.08%	(−0.20; 0.04)	0.175	−0.91%	(−2.50; 0.68)	0.263
6–8	26.8	−0.05%	(−2.81; 2.72)	0.974	−0.01%	(−2.62; 2.60)	0.996	−0.01%	(−0.15; 0.13)	0.901	−0.08%	(−1.96; 1.80)	0.933

**Table 4:** Association between physical activity and percent body composition measures and anthropometric measures among 142 men from the EPIC-Florence cohort.

	Increase by +1 MET Hours/Week			Highest vs. Lowest Quantile ^(a)^			
	β	95% CI	*p*-Value	β	95% CI	*p*-Value	*p*-Value for Trend
	**Walking**						
**Fat mass, %**	−0.03%	(−0.10; 0.04)	0.472	−2.40%	(−5.94; 1.13)	0.182	0.569
**Muscle mass, %**	0.02%	(−0.04; 0.09)	0.479	2.30%	(−0.99; 5.59)	0.171	0.558
**Bone mass, %**	0.002%	(−0.002; 0.006)	0.262	0.191%	(0.020; 0.362)	0.029	0.247
**Visceral fat, score**	−0.02	(−0.06; 0.02)	0.378	−1.93	(−3.94; 0.09)	0.061	0.320
**Total body water, %**	0.03%	(−0.02; 0.08)	0.230	2.29%	(−0.08; 4.66)	0.058	0.282
**Body mass index, kg/m^2^**	−0.02	(−0.06; 0.03)	0.536	−1.57	(−3.96; 0.83)	0.198	0.474
**Waist circumference, cm**	−0.09	(−0.23; 0.05)	0.192	−6.69	(−13.57; 0.18)	0.056	0.128
	**Fitness**						
**Fat mass, %**	−0.08%	(−0.14; −0.02)	**0.014**	−3.29%	(−5.65; −0.93)	**0.006**	**0.008**
**Muscle mass, %**	0.07%	(0.02; 0.13)	**0.013**	3.10%	(0.89; 5.32)	**0.006**	**0.007**
**Bone mass, %**	0.004%	(0.001; 0.007)	**0.010**	0.176%	(0.055; 0.297)	**0.004**	**0.004**
**Visceral fat, score**	−0.04	(−0.08; −0.01)	**0.014**	−1.99	(−3.37; −0.61)	**0.005**	**0.004**
**Total body water, %**	0.05%	(0.01; 0.10)	**0.010**	2.11%	(0.47; 3.75)	**0.012**	**0.014**
**Body mass index, kg/m^2^**	−0.05	(−0.09; −0.01)	**0.024**	−2.31	(−3.94; −0.69)	**0.006**	**0.004**
**Waist circumference, cm**	−0.16	(−0.28; −0.05)	**0.007**	−7.24	(−11.92; −2.56)	**0.003**	**0.002**
	**Recreational physical activity**						
**Fat mass, %**	−0.04%	(−0.07; −0.01)	**0.029**	−2.09%	(−4.87; 0.70)	0.142	0.065
**Muscle mass, %**	0.03%	(0.00; 0.07)	**0.028**	1.97%	(−0.64; 4.57)	0.139	0.067
**Bone mass, %**	0.002%	(0.000; 0.004)	**0.014**	0.133%	(−0.008; 0.273)	0.065	**0.039**
**Visceral fat, score**	−0.02	(−0.04; −0.00)	**0.030**	−1.34	(−2.97; 0.29)	0.107	**0.049**
**Total body water, %**	0.03%	(0.01; 0.05)	**0.012**	1.45%	(−0.46; 3.36)	0.138	0.078
**Body mass index, kg/m^2^**	−0.02	(−0.05; −0.00)	**0.042**	−1.58	(−3.49; 0.034)	0.105	**0.046**
**Waist circumference, cm**	−0.09	(−0.16; −0.03)	**0.004**	−5.36	(−10.87; 0.16)	0.057	**0.025**
	**Household physical activity**						
**Fat mass, %**	−0.01%	(−0.05; 0.03)	0.624	−0.74%	(−3.51; 2.04)	0.604	0.402
**Muscle mass, %**	0.01%	(−0.03; 0.05)	0.587	0.80%	(−1.80; 3.40)	0.547	0.363
**Bone mass, %**	0.000%	(−0.002; 0.003)	0.697	0.045%	(−0.096; 0.186)	0.532	0.384
**Visceral fat, score**	0.00	(−0.03; 0.02)	0.782	−0.57	(−2.20; 1.06)	0.487	0.371
**Total body water, %**	0.01%	(−0.02; 0.04)	0.350	0.83%	(−1.07; 2.73)	0.393	0.249
**Body mass index, kg/m^2^**	0.00	(−0.03; 0.03)	0.817	−0.24	(−2.16; 1.68)	0.803	0.648
**Waist circumference, cm**	−0.04	(−0.13; 0.04)	0.339	−1.23	(−6.76; 4.30)	0.660	0.452

^(a)^ 4th vs. 1st quartile for walking, recreational physical activity, and household physical activity and 3rd vs. 1st tertile for fitness.

**Table 5:** Association between physical activity and percent body composition measures and anthropometric measures among 183 women from the EPIC-Florence cohort.

	Increase by +1 MET Hours/Week			Highest vs. Lowest Quantile ^(a)^			
	β	95% CI	*p*-Value	β	95% CI	*p*-Value	*p*-Value for Trend
	**Walking**						
**Fat mass, %**	−0.04%	(−0.13; 0.05)	0.378	−1.69%	(−4.48; 1.10)	0.236	0.374
**Muscle mass, %**	0.04%	(−0.05; 0.13)	0.377	1.62%	(−1.01; 4.25)	0.227	0.362
**Bone mass, %**	0.002%	(−0.003; 0.006)	0.456	0.091%	(−0.049; 0.231)	0.203	0.344
**Visceral fat, score**	−0.02	(−0.05; 0.01)	0.251	−0.10	(−1.97; 0.08)	0.071	0.144
**Total body water, %**	0.03%	(−0.03; 0.09)	0.380	1.23%	(−0.66; 3.11)	0.202	0.322
**Body mass index, kg/m^2^**	−0.05	(−0.11; 0.02)	0.182	−2.01	(−4.04; 0.01)	0.051	0.118
**Waist circumference, cm**	−0.10	(−0.26; 0.07)	0.245	−4.32	(−9.28; 0.64)	0.088	0.227
	**Fitness**						
**Fat mass, %**	−0.12%	(−0.18; −0.05)	**0.000**	−3.72%	(−6.02; −1.41)	**0.002**	**0.001**
**Muscle mass, %**	0.11%	(0.05; 0.17)	**0.000**	3.50%	(1.33; 5.67)	**0.002**	**0.001**
**Bone mass, %**	0.006%	(0.003; 0.009)	**0.000**	0.201%	(0.083; 0.318)	**0.001**	**0.001**
**Visceral fat, score**	−0.04	(−0.06; −0.02)	**0.001**	−1.43	(−2.28; −0.58)	**0.001**	**0.001**
**Total body water, %**	0.08%	(0.03; 0.12)	**0.000**	2.54%	(0.97; 4.11)	**0.002**	**0.001**
**Body mass index, kg/m^2^**	−0.08	(−0.12; −0.03)	**0.001**	−2.85	(−4.53; −1.17)	**0.001**	**0.001**
**Waist circumference, cm**	−0.15	(−0.26; −0.03)	**0.012**	−6.02	(−10.17; −1.87)	**0.005**	**0.004**
	**Recreational physical activity**						
**Fat mass, %**	−0.08%	(−0.13; −0.04)	**0.000**	−3.59%	(−6.37; −0.81)	**0.011**	**0.004**
**Muscle mass, %**	0.08%	(0.04; 0.12)	**0.000**	3.43%	(0.82; 6.05)	**0.010**	**0.004**
**Bone mass, %**	0.004%	(0.002; 0.006)	**0.000**	0.180%	(0.039; 0.321)	**0.012**	**0.005**
**Visceral fat, score**	−0.03	(−0.04; −0.01)	**0.000**	−1.57	(−2.59; −0.54)	**0.003**	**0.001**
**Total body water, %**	0.06%	(0.03; 0.08)	**0.000**	2.57%	(0.68; 4.45)	**0.008**	**0.003**
**Body mass index, kg/m^2^**	−0.05	(−0.08; −0.02)	**0.000**	−2.80	(−4.81; −0.79)	**0.007**	**0.001**
**Waist circumference, cm**	−0.09	(−0.17; −0.02)	**0.010**	−5.27	(−10.30; −0.24)	**0.040**	**0.017**
	**Household physical activity**						
**Fat mass, %**	−0.01%	(−0.04; 0.02)	0.465	0.18%	(−2.66; 3.01)	0.903	0.990
**Muscle mass, %**	0.01%	(−0.02; 0.03)	0.476	−0.17%	(−2.84; 2.50)	0.900	0.990
**Bone mass, %**	0.000%	(−0.001; 0.002)	0.465	−0.008%	(−0.151; 0.135)	0.912	0.924
**Visceral fat, score**	0.00	(−0.01; 0.01)	0.569	−0.08	(−1.13; 0.98)	0.885	0.747
**Total body water, %**	0.01%	(−0.01; 0.02)	0.503	−0.12%	(−2.04; 1.80)	0.902	0.998
**Body mass index, kg/m^2^**	0.00	(−0.02; 0.02)	0.667	−0.18	(−2.26; 1.90)	0.866	0.696
**Waist circumference, cm**	−0.01	(−0.06; 0.03)	0.583	0.10	(−4.98; 5.18)	0.969	0.850

^(a)^ 4th vs. 1st quartile for walking, recreational physical activity, and household physical activity and 3rd vs. 1st tertile for fitness.

## Data Availability

The data presented in this study are available on request from the corresponding author due to privacy reasons.

## Abbreviations

The following abbreviations are used in this manuscript:

BIA	Bioelectrical Impedance Analysis
BMI	Body Mass Index
DASH	Dietary Approaches to Stop Hypertension
EPIC	European Prospective Investigation into Cancer and Nutrition
FCT	Food Composition Tables
FFQ	Food Frequency Questionnaire
GMMD	Greek Modified Mediterranean Diet
IMI	Italian Mediterranean Index
LSQ	Lifestyle Questionnaire
MET	Metabolic Equivalent
PA	Physical Activity
PPAR-c	Peroxisome Proliferator-Activated Receptor-C
SD	Standard Deviation
WC	Waist Circumference
